# Supplementary material for: The Gene Expression Profile of the Song Control Nucleus HVC Shows Sex Specificity, Hormone Responsiveness, and Species Specificity Among Songbirds
Source: Front Neurosci. 2021 May 31;15:680530. doi: 10.3389/fnins.2021.680530 (PMC8200640; doi:10.3389/fnins.2021.680530)
Supplement: Supplementary file 8 [file Data_Sheet_1.docx]

Supplementary Material

# Supplementary Tables and Figures

## Supplementary Table Legends

Supplementary Table 1. Fisher’s exact tests of sex-biased genes for chromosome enrichment.

Supplementary Table 2. GO term enrichment analysis of sex-biased genes identified from the forest weaver (comparison 1), cordon-bleu (comparison 2), and canary (comparison 5) comparisons.

Abbreviations: FWm, forest weaver male-biased genes; CBm, cordon-bleu male-biased genes; Cm, canary male-biased genes; FWf, forest weaver female-biased genes; CBm, cordon-bleu female-biased genes; CfS, canary female-biased genes.

Supplementary Table 3. Pearson’s correlation analysis of principal components and variables (plasma androgen levels, HVC volume, sex, and singing).

Supplementary Table 4. Female-specific expressed genes.

Abbreviations: SDfS: non-breeding spontaneously singing female canaries; SDfT: non-breeding testosterone-stimulated singing female canaries.

Supplementary Table 5. Male-specific expressed genes.

Abbreviations: LDm: breeding singing male canaries; SDmT: non-breeding testosterone-stimulated singing male canaries.

Supplementary Table 6. Sex-shared expressed genes.

Abbreviations: LDm: breeding singing male canaries; SDfS: non-breeding spontaneously singing female canaries; SDmT: non-breeding testosterone-stimulated singing male canaries; SDfT: non-breeding testosterone-stimulated singing female canaries.

Supplementary Table **7**. GO term enrichment analysis of female-specific, male-specific and sex-shared genes.

## Supplementary Figures


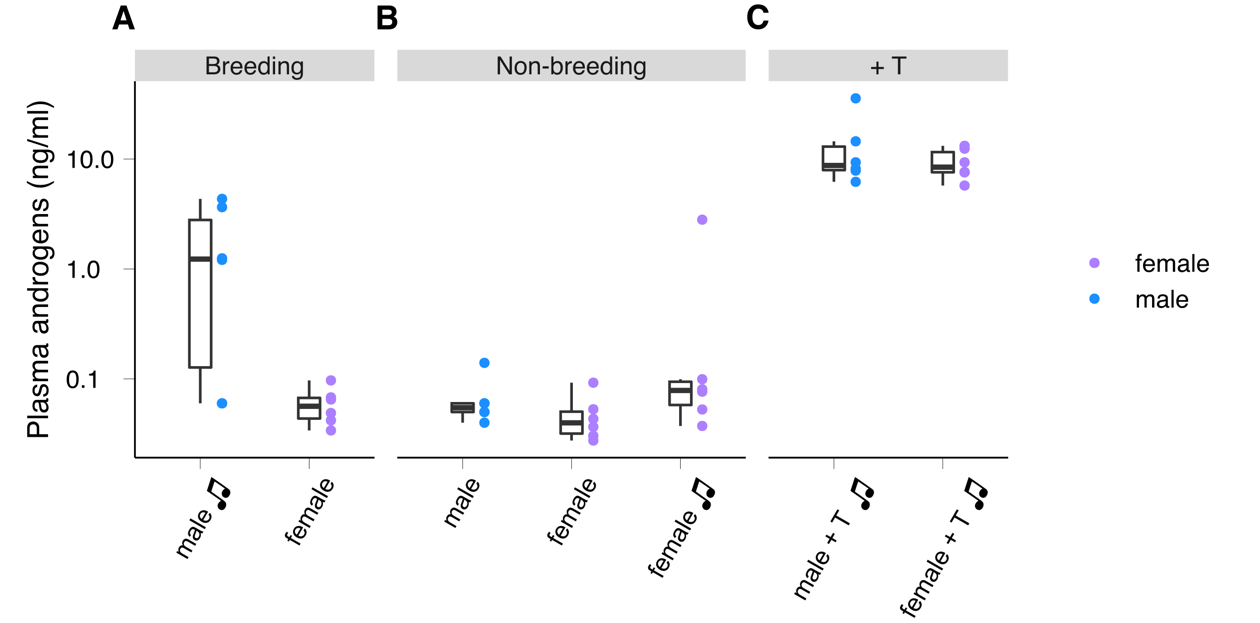


Supplementary Figure 1. Plasma androgen levels of seven groups of canaries on the day of sacrifice.

**A,** Canaries in breeding conditions. Singing males 1.76 ng/ml (mean), non-singing females 59.2 pg/ml. Mann-Whitney Test, U = 6, P value = 0.07. **B,** Canaries in non-breeding conditions. Non-singing males 66.7 pg/ml (mean), non-singing females 47.2 pg/ml, singing females 52.6 pg/ml. Mann-Whitney Test (non-singing males vs. non-singing females), U = 9, P value = 0.172. Mann-Whitney Test (non-singing males vs. singing females), U = 24, P value = 0.377. **C,** Testosterone-implanted non-breeding canaries. Males 13.5 ng/ml, females 9.31 ng/ml. Mann-Whitney Test, U = 13, P value = 0.485. Testosterone implantation significantly increased the plasma androgen levels of both non-breeding males and females Mann-Whitney Test (non-breeding non-singing males vs. testosterone-implanted singing males), U = 0, P value = 0.00492. Mann-Whitney Test (non-breeding non-singing females vs. testosterone-implanted singing females), U = 0, P value = 0.00217. The plasma androgen levels were higher in the breeding males than in the non-breeding non-singing males (Mann-Whitney Test, U = 32, P value = 0.0275). The boxes indicate the 25th/50th/75th percentiles (bottom/middle/top bar), and the extent of the whiskers indicates the most extreme values that are within 1.5 times the IQR (interquartile range) of the hinge. Each colour-coded dot indicates the measurement from one bird. The non-breeding singing female canaries data were obtained from Ko et al. (2020).


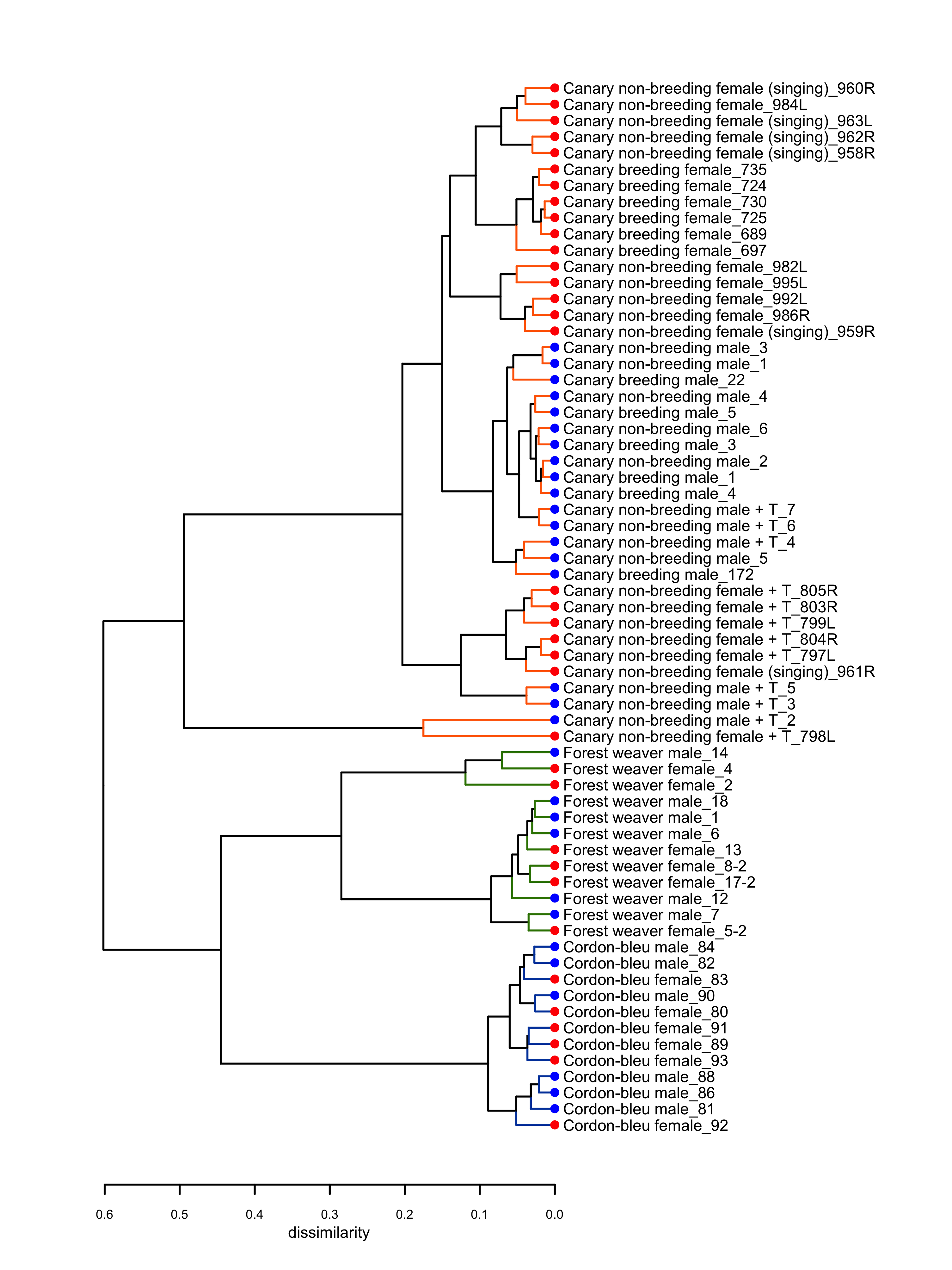


Supplementary Figure 2. Hierarchical clustering of the HVC transcriptomes of 65 birds used in this study.

Hierarchical clustering showed that the HVC transcriptomes were first clustered based on the phylogenetic relationship; among canaries, non-breeding females implanted with testosterone showed the most distinctive patterns. +T: testosterone implantation.


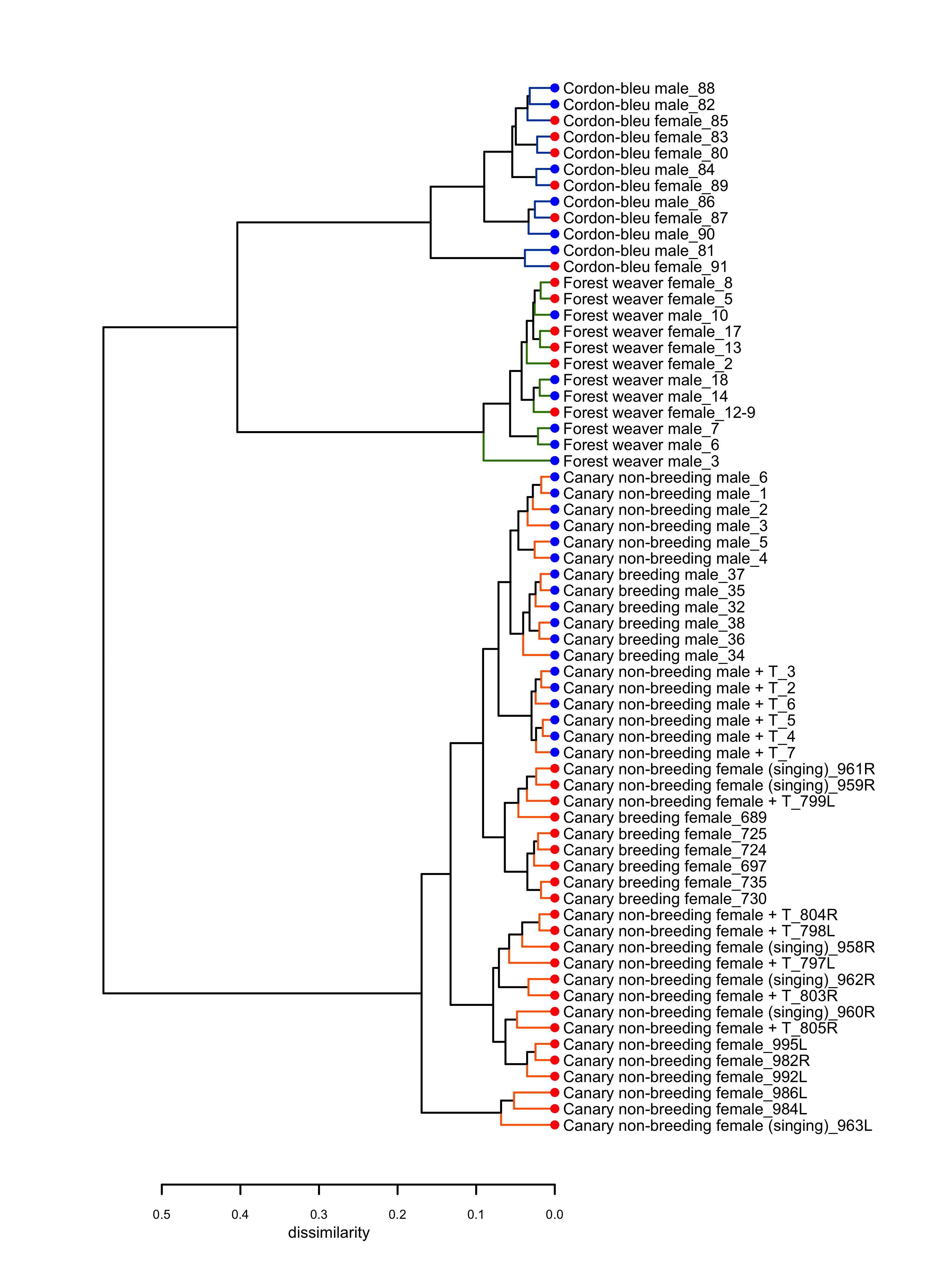


Supplementary Figure 3. Hierarchical clustering of the entopallium transcriptomes of 65 birds used in this study.

Hierarchical clustering showed that the entopallium transcriptomes were first clustered based on the phylogenetic relationship. The canaries were clustered by sex. +T: testosterone implantation.


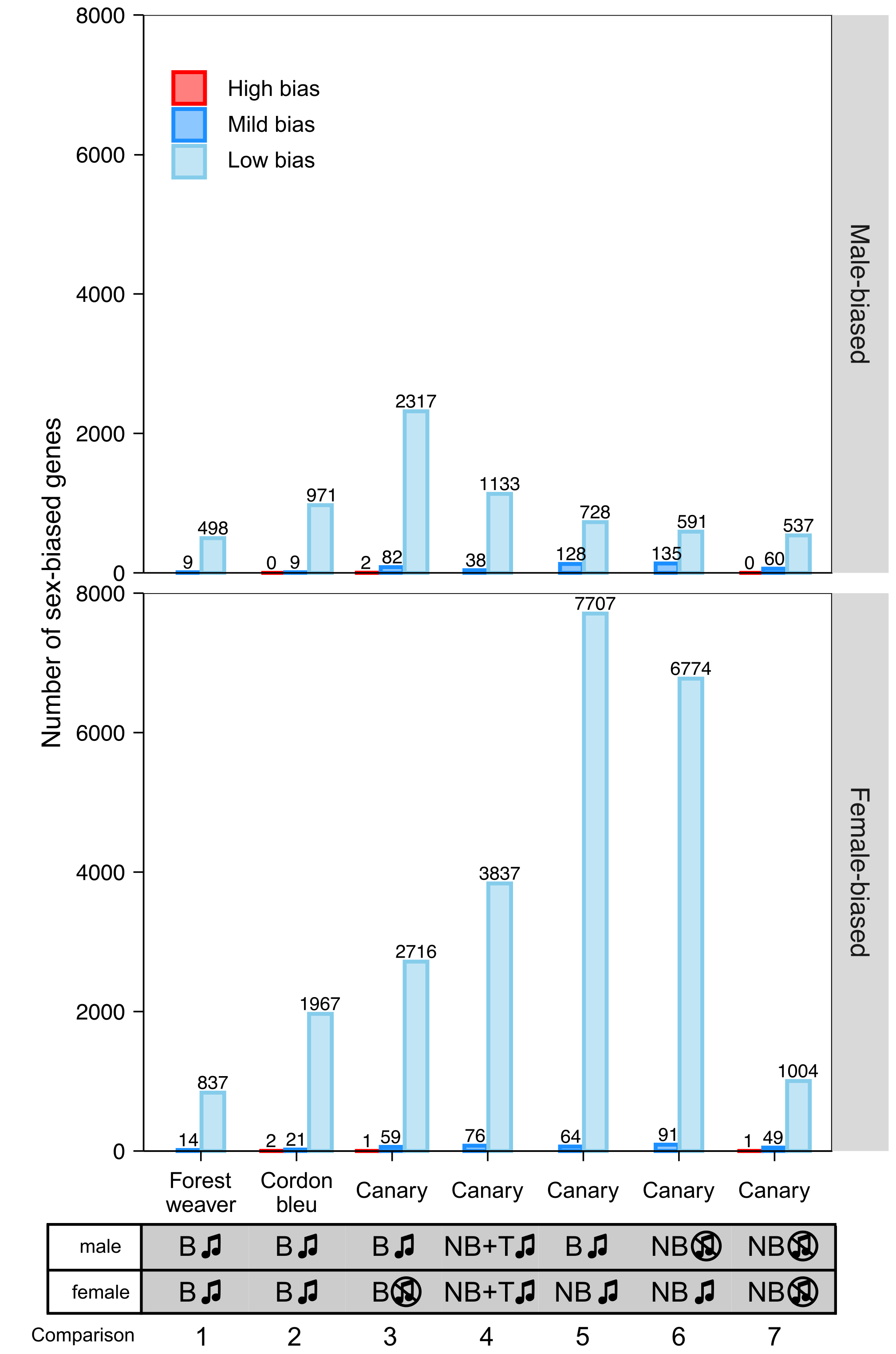


Supplementary Figure 4. The majority of sex-biased genes showed low sex bias.

The bar graph summarizes the number of sex-biased genes in the HVC transcriptome identified from each male-to-female comparison. High bias: |log_2_(fold change)| ≥ 2; moderate bias: 1 ≤ |log_2_(fold change)| < 2; low bias: 0.5 ≤ |log_2_(fold change)| < 1. The phenotypes of the groups being compared in each male-to-female comparison are listed at the bottom of the graph.


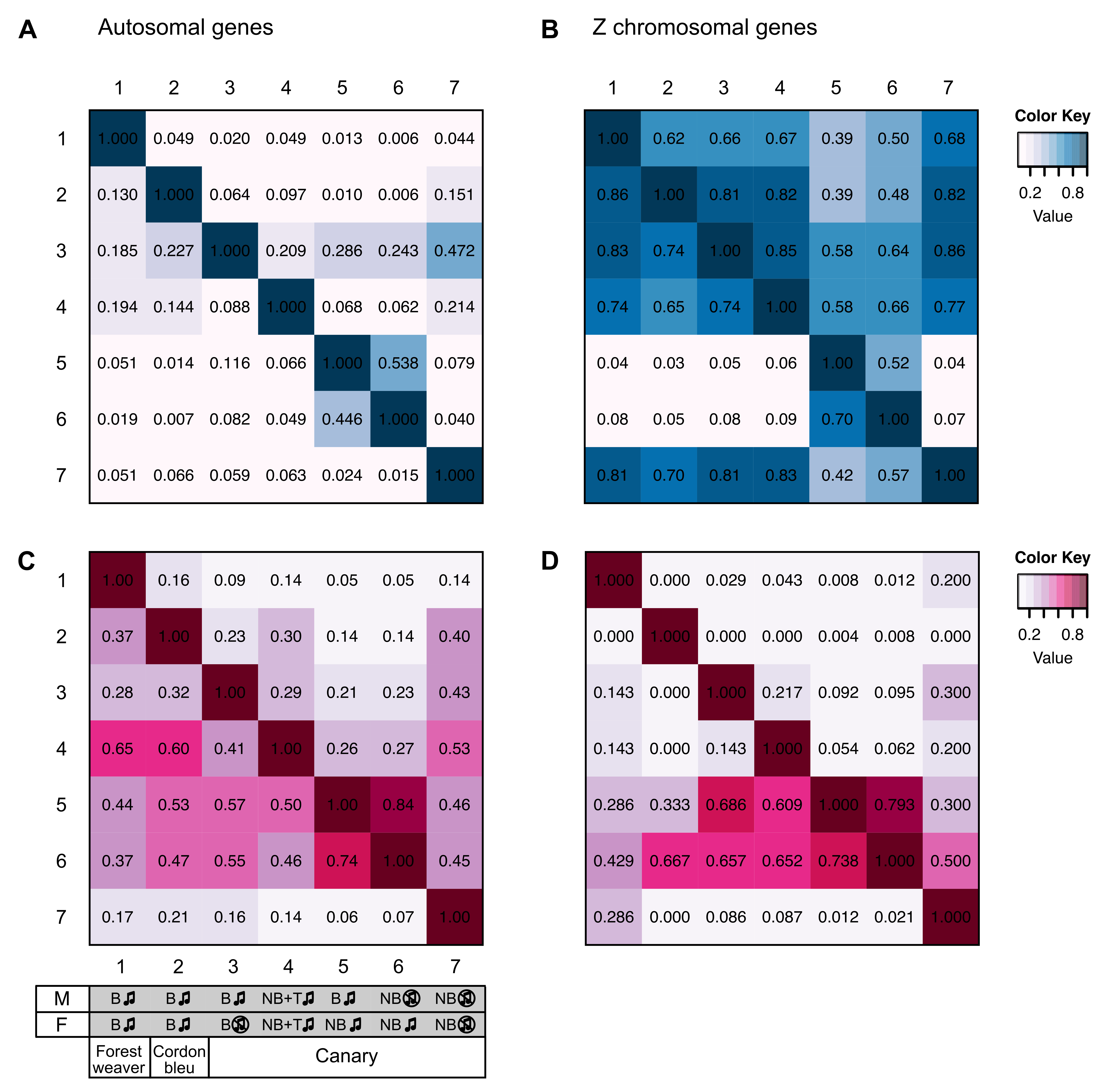


Supplementary Figure 5. Pairwise comparisons between autosomal and Z chromosomal sex-biased genes.

**A,** Autosomal male-biased genes. **B,** Z chromosomal male-biased genes. **C,** Autosomal female-biased genes. **D,** Z chromosomal female-biased genes. The numbers indicated in the matrices are the proportions of identical genes identified from each pair. The phenotypes of the groups used for each comparison are listed at the bottom. B: breeding; NB: non-breeding; T: testosterone implantation; the music note indicates the presence or absence of singing behaviour.


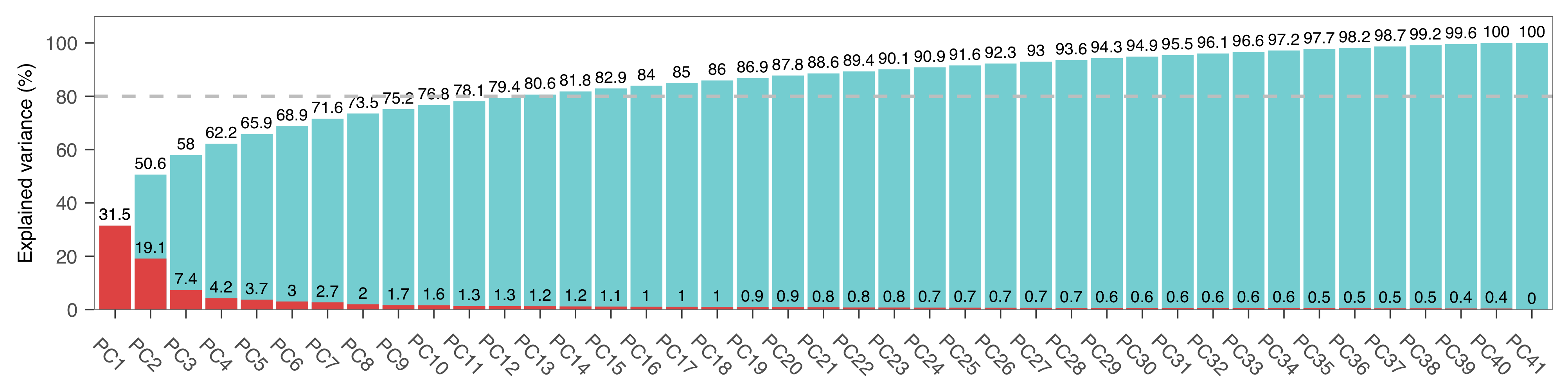


Supplementary Figure 6. PCA scree plot showing the percentage of variance explained by each principal component.
